# Very Low Birth Weight and Perinatal Periods of Risk: Disparities in St. Louis

**DOI:** 10.1155/2014/547234

**Published:** 2014-06-15

**Authors:** Pamela Xaverius, Joanne Salas, Deborah Kiel, Candice Woolfolk

**Affiliations:** ^1^Saint Louis University, College for Public Health & Social Justice, 3545 Lafayette Avenue, Saint Louis, MO 63104, USA; ^2^Lindenwood University, 209 S Kingshighway Street, St. Charles, MO 63301, USA

## Abstract

*Objective*. Very low birth weight (VLBW) is a significant issue in St. Louis, Missouri. Our study evaluated risk factors associated with VLBW in this predominantly urban community. *Methods*. From 2000 to 2009, birth and fetal death certificates were evaluated (*n* = 160, 189), and mortality rates were calculated for perinatal periods of risk. The Kitagawa method was used to explore fetoinfant mortality rates (FIMR) in terms of birth weight distribution and birthweight specific mortality. Multivariable logistic regression was used to assess the magnitude of association of selected risk factors with VLBW. *Results*. VLBW contributes to 50% of the excess FIMR in St. Louis City and County. The highest proportion of VLBW can be attributed to black maternal race (40.6%) in St. Louis City, inadequate prenatal care (19.8%), and gestational hypertension (12.0%) among black women. Medicaid was found to have a protective effect for VLBW among black women (population attributable risk (PAR) = −14.5). *Discussion*. Interventions targeting the health of women before and during conception may be most successful at reducing the disparities in VLBW in this population. Interventions geared towards smoking cessation and improvements in Medicaid and prenatal care access for black mothers and St. Louis City residents can greatly reduce VLBW rates.

## 1. Introduction

The health of the youngest and the smallest in our society is an important representation of the overall well-being of a population. Indeed, very low birth weight (VLBW) babies, less than 1500 grams, often represent the smallest and the newest members of our communities and are often at greatest risk for mortality and morbidity. While advances in medical care have increased the survival rates of VLBW infants, these infants are susceptible to neonatal complications, neurodevelopmental impairments, recurrent hospitalizations, chronic medical conditions, mental retardation, and learning disabilities [1, 2]. VLBW births also impact the families in which they occur [1, 2]. For example, mothers of VLBW infants have higher psychological distress and are more likely to experience depression and anxiety [2]. Healthy People 2020 recommends no more than 1.4 of every 1,000 births be VLBW [3]. In St. Louis City and County from 2000 to 2009, the overall VLBW rate was 2.0 per 1,000 live births, with 1.2 of 1,000 live births among whites born at VLBW and 3.3 of 1,000 live births among African Americans born at VLBW [4].

Due to the complex nature of infant morbidity and mortality, the perinatal periods of risk (PPOR) approach was established to provide a framework to investigate and prevent fetoinfant mortality in large, urban communities [5]. Fetoinfant deaths are sorted into four categories: maternal health/prematurity, maternal care, newborn care, and infant care, by birth weight and age at death. The categories roughly represent risk factors that can be used to guide interventions, specifically targeting the risk category with the greatest excess fetoinfant mortality in the target community in reference to a population that often has better birth outcomes. The elegance underpinning this approach, in our opinion, is the social-justice lens by which risk categories are prioritized and interventions are selected, with the overall goal of actionable change and improved infant health.

Previous PPOR analyses have found that the maternal health/prematurity category, which by definition consists of all VLBW births and fetal deaths, is often the greatest contributor to excess infant mortality [6–12]. A PPOR analysis in Kansas City found that deaths due VLBW were a significant issue among African Americans [7]. The Kitagawa analyses, which are used to determine the contributors to excess deaths in the maternal health/prematurity category, found that among black infants, 44.5% of the excess deaths were attributed to the distribution of VLBW births, compared to 24.3% among white infants in Kansas City [7]. Other PPOR analyses have identified risk factors for VLBW that include maternal medical risk, maternal anemia, obstetric history, black maternal race, maternal age less than 20 years or greater than 35 years, smoking, inadequate/no prenatal care, parity, maternal weight gain less than 11 pounds, socioeconomic status, and single marital status [2, 10, 13].

The greater St. Louis area is a unique location in the country with poorer birth outcomes than national averages and persistent disparities [14]. This present study is a PPOR analysis of St. Louis City and County using fetoinfant birth-death data from 2000 to 2009. This study examines disparities and excess fetoinfant mortality rates (FIMR) compared to a US referent group (2000–2002) in the context of person (black versus white), place (St. Louis City versus St. Louis County), and time (2000–2004 versus 2005–2009). We will explore excesses in FIMR by PPOR categories, compare birth weight distribution with birth-weight specific mortality, and assess population attributable risks associated with VLBW.

## 2. Materials and Methods

We conducted a multiyear, population-based cohort study (2000–2009) of birth, death, and fetal-death records for St. Louis City and County, using a PPOR framework. Before any restrictions, there were 174,558 records in the live birth file. Live birth records were excluded from the analysis if (1) they were missing birth weight data (36 or 0.02%), (2) birth weight was less than 500 grams (585; 0.34%), (3) race/ethnicity was not black or white, non-Hispanic (14,347 or 8.2%), and (4) they were implausible gestational age and birth weight combinations (119 or .07%), per PPOR analytic guidelines [5, 15]. After all exclusion criteria were applied, the final live birth file had 159,547 live birth records. Fetal deaths records were also included in the analysis, with 1,366 records in the fetal death file for the years 2000–2009. Fetal death records were excluded if (1) they were missing birth weight (44 or 3.22%), (2) they were missing gestational age (3 or 0.22%), (3) birth weight was less than 500 grams (536 or 39.2%), (4) gestational age was less than 24 weeks (505 or 37.0%), (5) race/ethnicity was not black or white, non-Hispanic (106 or 7.8%), and (6) they were implausible gestational age and birth weight combinations (25 or 1.8%). After all exclusion criteria were applied, final fetal death record sample size was 642.

Fetoinfant deaths were distributed into four categories by birth weight (500–1499 g, ≥1500 g) and age at death (fetal [>24 weeks gestation and before birth], neonatal [birth day-27 days], and postneonatal [28–365 days old]). The maternal health/prematurity (MHP) category includes all birth weights of 500–1499 g (VLBW), independent of age at death. The maternal care (MC) category includes birth weights ≥1500 g and fetal deaths; the neonatal care (NC) category includes birth weights ≥1500 g and neonatal deaths; and the infant health (IH) category includes birth weights ≥1500 g and postneonatal deaths.

Covariates of interest included race (white, black), maternal age (≤20, 21–34, and >34), maternal education (<13, ≥13 years), Medicaid (yes, no), and parity (nulliparous, primiparous, and multiparous). Self-reported pregnancy risk factors included gestational hypertension (yes, no), chronic hypertension (yes, no), eclampsia (yes, no), chronic diabetes (yes, no), smoking status during pregnancy (yes, no), and inadequate prenatal care (yes, no). Inadequate prenatal care was defined as fewer than five prenatal visits for pregnancies less than 37 weeks gestation, fewer than eight visits for pregnancies 37 weeks gestation or more, or care beginning after the first four months of pregnancy.

The analytic plan included calculating FIMR rates for each risk category and calculating excess rates in comparison to a US national reference group [6]. An external reference group was used and included all white, non-Hispanic women residing in the United States, greater than 20 years of age, with more than 13 years of education from 2000 to 2002 [16]. The excess death rates per 1000 live births and fetal deaths in each category were multiplied by the total number of live births and fetal deaths in that category to yield the number of excess deaths, which were subsequently used to calculate the percentage contribution of each category to the number of excess deaths. The Kitagawa method was used to explore excess in birth weight distribution (i.e., higher frequency of prematurity) and birth weight specific mortality (i.e., higher mortality rates once born at a specific birth weight), using the same reference population.

Target populations used in the analysis were (1) entire target population of St. Louis City and County of all races, in 2000–2009; (2) black race, St. Louis City and County, in 2000–2009; and (3) St. Louis City, all races, in 2000–2009. This analysis was used to explain differences in FIMRs for the target groups, compared to the US reference groups, in terms of birth weight distribution and birth weight specific mortality.

A chi-square test at alpha = 0.05 assessed whether VLBW risk factors varied by race or county. Multivariable logistic regression was used to assess the magnitude of association of selected risk factors with VLBW in St. Louis City and County. Two separate multivariable models were calculated: (1) for St. Louis City and (2) for black women. Population attributable risk percentages (PAR%) were also calculated for each risk factor because communities or subpopulations vary according to risk factor distribution. Using the adjusted odds ratio for each risk factor and the prevalence of the risk factor in the target community of interest, PAR% was calculated as follows: PAR% = *P* (odds ratio [OR] − 1)/[*P* (OR − 1) + 1], where *P* equals the prevalence of the risk factor. The PAR% represents the proportion of VLBW that can be attributed to the risk factor of interest. If PAR% is negative (i.e., indicates a protective factor), PAR% becomes difficult to interpret but roughly indicates the proportion increase in VLBW in the population if the protective factor were not present. PAR% is useful to aid in prioritizing intervention and prevention efforts because it is an estimate of the factor's effect on the outcome. A higher positive PAR% indicates that a higher proportion of VLBW can be attributable to the risk factor of interest.

## 3. Results

FIMRs as well as excess rates and deaths compared to the US referent group are presented in Table 1. The overall FIMR for St. Louis City and County in 2000 to 2009 was 10.3 per 1,000 live births and fetal deaths. The FIMR was 13.0 per 1,000 live births and fetal deaths in St. Louis City, 9.1/1000 live births and fetal deaths in St. Louis County, 16.4 per 1,000 live births and fetal deaths among black women, and 5.8 per 1,000 live births and fetal deaths in white women. Compared to the 2000 to 2002 US referent FIMR of 5.7 per 1,000 live births and fetal deaths, St. Louis City and County had an excess FIMR of 4.6 per 1,000 live births and fetal deaths, translating to a total of 731 excess fetoinfant deaths from 2000 to 2009. The excess FIMR was 7.3 per 1,000 live births and fetal deaths in St. Louis City and 10.7 per 1,000 live births and fetal deaths in black women, compared to the national average. The PPOR analysis identified MHP as the largest contributor to excess fetoinfant deaths in St. Louis City and County from 2000 to 2009. FIMRs were fairly similar comparing 2000 to 2004 (10.8 per 1,000 live births and fetal deaths) and 2005 to 2009 (9.7 per 1,000 live births and fetal deaths). The MHP category contributed the most to excess fetoinfant deaths (50%), followed by IH (28%), MC (20%), and NC (2%).

The results of the Kitagawa analysis for the categories with the greatest excess death (i.e., black, non-Hispanic, and St. Louis City) and for the total target population are presented in Table 2. For the total target population (All), birth weight distribution accounts for 63.6% of the overall excess FIMR when compared to the US reference group. Among VLBW births (MHP category), 48.3% of the excess mortality can be attributed to birth weight distribution compared to 3.2% attributable to birth weight specific mortality. Results for the St. Louis City and black populations were similar, with overall 59.7% of excess deaths for blacks and 59.8% excess in St. Louis City due to birth weight distribution. These results show that there is a large number of LBW babies being born, VLBW babies in particular, so further analysis focused on evaluating risk factors for VLBW is necessary. Also, results of Tables 1 and 2 show that the populations with the greatest burden of excess FIMR are St. Louis City and black women. Thus, an examination of these risk factors among these populations is warranted.

The prevalence of selected risk factors for VLBW for the total target population by race and by county is presented in Table 3. Table 3 shows that there were significant racial and geographic disparities in risk factors for VLBW. For example, 70.4% of black women compared to 19.5% of white women were on Medicaid (*P* < .0001). Similarly, more city residents were on Medicaid than county residents (62.9% versus 31.3%). Slightly more black women (12.2%) and city residents (16.2%) smoked during pregnancy compared to white (11.1%) and county residents (9.5%), respectively. Compared to white women, black women were also more likely to have inadequate prenatal care, diabetes, chronic hypertension, gestational hypertension, and eclampsia. Similar results, except for eclampsia and diabetes, were found when comparing St. Louis City to County.

Adjusted odds ratios (aOR) and PAR% for risk factors associated with VLBW in St. Louis City are presented in Table 4. In St. Louis City, black maternal race, maternal age > 34, inadequate prenatal care, smoking, PIH, and eclampsia were associated with significantly higher odds of VLBW from 2000 to 2009. Black maternal race was associated with 2.04 times the odds of VLBW (95% CI: 1.69, 2.45), while maternal age > 34 was associated with a 51% increase in the odds of VLBW (aOR: 1.51; 95% CI: 1.22, 1.87). Inadequate prenatal care was associated with 2.12 times the odds of VLBW (95% CI: 1.84, 2.45). Smoking was associated with an 18% increase in the odds of VLBW (aOR: 1.18; 95% CI: 1.01, 1.40). Gestational hypertension was associated with 3.28 times the odds of VLBW (95% CI: 2.77, 3.88). Based on the PAR%, the highest proportion of VLBW in St. Louis City can be attributed to black maternal race (PAR%: 40.56), followed by inadequate prenatal care (PAR%: 17.32).

Adjusted odds ratios and PAR% for risk factors associated with VLBW for black women for St. Louis City and County are presented in Table 5. Among black women, maternal age > 34, Medicaid, inadequate prenatal care, nulliparity, smoking, chronic hypertension, gestational hypertension, and eclampsia were significantly associated with VLBW. Inadequate prenatal care was associated with 2.19 times the odds of VLBW (95% CI: 1.96, 2.44). Nulliparity was associated with a 15% increased odds of VLBW (aOR: 1.15; 95% CI: 1.01, 1.31). Smoking was associated with a 25% increased odds of VLBW (aOR: 1.25; 95% CI: 1.08, 1.43). Gestational hypertension was associated with 2.89 times the odds of VLBW (95% CI: 2.54, 3.28). Medicaid was associated with an 18% decreased odds of VLBW (aOR: 0.82; 95% CI: 0.73, 0.91). Based on the PAR%, the highest proportion of VLBW among black infants can be attributed to inadequate prenatal care (PAR%: 19.84), followed by gestational hypertension (PAR%: 11.98). Medicaid was found to have a large protective effect; VLBW births would increase by approximately 14.5% if black mothers were not on Medicaid compared to mothers who were on Medicaid.

## 4. Discussion

From 2000 to 2009, there was an excess FIMR of 4.6 per 1,000 live births and fetal deaths, representing an excess of 731 fetoinfant deaths in St. Louis City and County, compared to the national reference group. The highest excess FIMRs were observed among black, non-Hispanic women and in St. Louis City. The FIMR in black, non-Hispanics was 16.4 per 1,000 live births and fetal deaths, almost three times the rate observed in the national reference group. In the total sample, 50% of excess fetoinfant deaths were associated with the MHP category and 28% of fetoinfant deaths were associated with the IH category. While all previous studies have identified MHP as the major contributor to excess fetoinfant deaths, the second highest contributor varies between maternal care and infant health, depending on community [6–9, 11].

Temporal changes in infant mortality rates were examined by comparing fetoinfant deaths from 2000 to 2004 and 2005 to 2009 for the total sample. FIMR dropped slightly from 10.8 to 9.7 per 1000 live births and fetal deaths from 2000 to 2004 and 2005 to 2009. The percentage of excess fetoinfant deaths associated with the MHP slightly increased as well from 47% in 2000–2004 to 52% in 2005–2009. Infant health contributed to increase from 26% to 32% in post neonatal mortality. The percentage of excess fetoinfant deaths associated with MC and NC decreased over time (24% to 12% and 4% to 2%, resp.). However, a temporal PPOR analysis conducted in Kansas City found that excess deaths due to MHP and IH decreased over time, while excess deaths due to MC and NC increased or remained the same [9]. These results in this analysis show that MHP and IH, particularly MHP because of its greater relative contribution to excess FIMR, are becoming more of an issue in this community. To address this increasing issue, interventions geared towards improving the health behaviors of mothers and perinatal care are essential.

Analyses were conducted to determine the contributors to excess FIMR. The Kitagawa analysis shows that the majority of excess deaths among VLBW infants are due to birth weight distribution, meaning that a disproportionately large number of VLBW babies are being born in this community. Therefore, interventions designed to reduce the number of VLBW infants can help to reduce excess fetoinfant mortality in this community. Due to a greater proportion of excess FIMR due to birth weight distribution and the fact that St. Louis City and black women carried the largest burden of excess FIMR, we examined risk factors for VLBW for St. Louis City and County and for African Americans. In St. Louis City, black maternal race, maternal age >34, inadequate prenatal care, smoking, gestational hypertension, and eclampsia were associated with significantly higher odds of VLBW. Among black mothers, maternal age > 34, Medicaid, inadequate prenatal care, nulliparity, smoking, and chronic hypertension are associated with VLBW. A previous PPOR analysis conducted in Kansas City identified maternal medical risk as the only significant risk factor of VLBW [7]. A PPOR analysis in Colorado identified maternal age < 20, marital status, and weight gain < 11 pounds as risk factors [10]. However, marital status and weight gain were not evaluated in our analysis; and maternal age < 20 was not associated with an increased risk of VLBW. Rather, maternal age > 34 compared to 21 to 34 was associated with an increased odds of VLBW. The biggest contributor to VLBW among blacks is inadequate prenatal care. Interventions geared towards improving access to prenatal care would be beneficial for black mothers. A high proportion of VLBW among black infants is also attributable to gestational hypertension. While the etiology of gestational hypertension is not well understood, our community should focus on secondary prevention methods, such as the early identification and clinical management of gestational hypertension when it occurs. Medicaid was found to be protective against VLBW among black women. The results suggest that if more black women in this community were on Medicaid, the number of VLBW births would decrease. Thus, this population may benefit from aggressive Medicaid enrollment.

This study has a few limitations. Generalizability is limited because this study only included data from black, non-Hispanics and white, non-Hispanics residing in St. Louis City and County. Results of this study may not be applicable to other races and geographical areas. Adequacy of prenatal care was assessed based on the number of prenatal visits. A recent study examining the relationship between prenatal care and preterm birth found that the content and timing of prenatal care was associated with preterm birth; there was no association between the number of visits and preterm birth [17]. This suggests that the content of prenatal care may be a more accurate measure for adequacy of prenatal care. Furthermore, mothers who deliver preterm infants may not have the opportunity to engage in as many prenatal visits, due to the short length of pregnancy. Medicaid was protective against VLBW in this analysis. However, we lacked information on other types of health insurance. Additionally, the US reference group used data from 2000 to 2002, but it was compared to data from 2000 to 2009. The US reference data could have changed over time. Birth certificate data and fetal death reports were used, and there is a possibility of inaccurate reporting. Previous studies have found that tobacco use, prenatal care visits, and weight gain during pregnancy are less accurately reported on birth and fetal death certificates [18, 19]. Residual confounding is another limitation of this study. We lacked information on a number of potential confounders, like substance abuse, which could not be accounted for and could have possibly affected the results. There is also a possibility of self-report bias and recall bias because all covariates were self-reported.

In spite of these limitations, this analysis has important strengths, notwithstanding the focus of this PPOR analysis in the St. Louis community, where poor birth outcomes and significant disparities persist. Because VLBW is the greatest contributor to excess fetoinfant mortality, interventions targeting the health of women before and during conception may be most successful in this community. A disproportionately large number of VLBW infants are born in this community which ultimately increases FIMRs. Interventions focused on reducing the number of VLBW infants can ultimately reduce fetoinfant mortality in the St. Louis community. The results also show disparities in FIMRs by race and area of residence. Interventions geared towards gestational hypertension prevention and treatment and improvements in prenatal care access for black mothers and St. Louis City residents can greatly reduce the number of VLBW black infants. Future studies could examine predictors and barriers to adequate prenatal care in this population. Analysis of temporal changes in infant mortality rates in the St. Louis community shows that deaths due to maternal health and prematurity and infant health are increasing slightly. Therefore, improvements in maternal health and education, prematurity, and access to care, smoking cessation programs, and more aggressive Medicaid enrollment are needed in this community.

## Figures and Tables

**Table 1:** Fetal-Infant Mortality Rates (FIMR; *per 1000 live births and fetal deaths*) and excess deaths by risk period and population characteristics, St. Louis City and County, Missouri, 2000–2009.

	FIMR					Numbers	
	Maternal Health/Prematurity	Maternal Care	Newborn Care	Infant Health	Total	Feto-infant deaths	Fetal deaths-live births
All	4.5	2.4	1.2	2.2	10.3	1,644	160,189
Time							
2000–2004	4.6	2.7	1.3	2.2	10.8	880	81,529
2005–2009	4.3	2.0	1.2	2.2	9.7	764	78,660
County							
St. Louis County	4.0	2.0	1.1	1.9	9.1	1,009	111,462
St. Louis City	5.5	3.1	1.4	3.0	13.0	635	48,727
Race							
White	2.3	1.4	0.9	1.2	5.8	536	92,696
Black	7.4	3.7	1.7	3.6	16.4	1,108	67,493
US Reference, 2000–2002	2.2	1.5	1.1	0.9	5.7		

Excess deaths versus US Reference, 2000–2002							
All	2.3	0.9	0.1	1.3	4.6	731	160,189
2000–2004	2.4	1.2	0.2	1.3	5.1	415	81,529
2005–2009	2.1	0.5	0.1	1.3	4.0	316	78,660
St. Louis County	1.8	0.5	0.0	1.0	3.4	374	111,462
St. Louis City	3.3	1.6	0.3	2.1	7.3	357	48,727
White	0.1	−0.1	−0.2	0.3	0.1	8	92,696
Black	5.2	2.2	0.6	2.7	10.7	723	67,493

**Table 2:** Kitagawa analysis, percentage contribution to differences in excess fetal-infant mortality rates, St. Louis City and County, Missouri, 2000–2009*.

PPOR Category	PA to birth weight distribution (%)	PA to birth weight specific mortality rates (%)	Total (%)
Maternal Health/Prematurity			
Black, non-Hispanic	46.1	3.4	49.5
St. Louis City	43.8	2.8	46.7
All	48.3	3.2	51.5
Maternal Care, Newborn Care, and infant death			
Black, non-Hispanic	13.6	36.9	50.5
St. Louis City	16.0	37.4	53.4
All	15.3	33.2	48.5
Total (All categories)			
Black, non-Hispanic	59.7	40.3	100
St. Louis City	59.8	40.2	100
All	63.6	36.4	100

*PA = Percent attributable.

**Table 3:** Prevalence of risk and preventive factors for VLBW among all live births/fetal deaths, St. Louis City and County, 2000–2009 (*N* = 160,189).

Risk/Preventive Factors	Overall	Race			County		
	Total Population	Black	White	*P*-value	St. Louis County	St. Louis City	*P*-value
	(*N* = 160,189)	(*n* = 67,493)	(*n* = 92,696)		(*n* = 111,462)	(*n* = 48,727)	
	%	%	%		%	%	
Maternal Age				<.0001			<.0001
<20	11.1	20.3	4.5		8.3	17.6	
21–34	74.2	72.2	75.6		74.6	73.2	
>34	14.7	7.5	19.9		17.2	9.2	
Maternal Education				<.0001			<.0001
<13 years	41.5	65.0	24.6		32.8	61.6	
13 + years	58.5	35.0	75.4		67.2	38.4	
Medicaid (yes)	40.9	70.4	19.5	<.0001	31.3	62.9	<.0001
Inadequate Prenatal Care (yes)	10.8	20.8	3.8	<.0001	7.4	18.7	<.0001
Parity				<.0001			<.0001
Nulliparous	40.5	37.3	42.9		40.5	40.6	
Primiparous	29.8	26.5	32.2		31.3	26.3	
Multiparous	29.7	36.2	24.9		28.2	33.1	
Smoking (yes)	11.5	12.2	11.1	<.0001	9.5	16.2	<.0001
Diabetes (yes)	3.9	4.1	3.8	<.0001	3.9	3.9	.42
Chronic Hypertension (yes)	1.8	2.7	1.0	<.0001	1.6	2.1	<.0001
Gestational Hypertension (yes)	5.7	7.2	4.6	<.0001	5.5	6.3	<.0001
Eclampsia (yes)	0.07	0.09	0.05	<.001	0.07	0.08	.46

**Table 4:** Adjusted odds ratios (AOR), 95% confidence intervals (CI), risk factor prevalence and population attributable risk percents (PAR%) for risk factors associated with very low birth weight in St. Louis City 2000–2009.

Risk/Preventive Factors	AOR	95% CI	Prevalence	PAR%
*St. Louis City *(*n* = 48727)				
Maternal Race: Black	**2.04**	**1.69, 2.45**	**0.66**	**40.56**
Maternal Age: <20	0.96	0.80, 1.16	0.18	−0.71
Maternal Age: >34	**1.51**	**1.22, 1.87**	**0.09**	**4.48**
Maternal Education: <13 years	1.09	0.93, 1.29	0.62	5.25
Medicaid: Yes	0.93	0.80, 1.09	0.63	−4.61
Inadequate Prenatal Care: Yes	**2.12**	**1.84, 2.45**	**0.19**	**17.32**
Parity: Nulliparous	1.04	0.87, 1.23	0.41	1.60
Parity: Multiparous	0.88	0.74, 1.04	0.33	−4.14
Smoking: Yes	**1.18**	**1.01, 1.40**	**0.16**	**2.83**
Diabetes: Yes	0.89	0.64, 1.25	0.04	−0.43
Chronic Hypertension: Yes	1.39	0.98, 1.97	0.02	0.81
Gestational Hypertension: Yes	**3.28**	**2.77, 3.88**	**0.06**	**12.56**
Eclampsia: Yes	**15.30**	**6.50, 36.02**	**0.001**	**1.13**

*Bolded risk factors are significant at *P* < .05.

**Table 5:** Adjusted odds ratios (AOR), 95% confidence intervals (CI), risk factor prevalence and population attributable risk percentages (PAR%) for risk factors associated with very low birth weight, by Black race, St. Louis City and County, 2000–2009 (*N* = 160,189).

Risk/Preventive Factors	AOR	95% CI	Prevalence	PAR%
*Black *(*n = 67493*)				
County: St. Louis City	0.96	0.87, 1.07	0.47	−1.93
Maternal Age: <20	0.87	0.76, 1.01	0.20	−2.71
Maternal Age: >34	**1.33**	**1.12, 1.57**	**0.08**	**2.42**
Maternal Education: <13 years	1.00	0.89, 1.12	0.65	0.00
Medicaid: Yes	**0.82**	**0.73, 0.91**	**0.70**	−**14.51**
Inadequate Prenatal Care: Yes	**2.19**	**1.96, 2.44**	**0.21**	**19.84**
Parity: Nulliparous	**1.15**	**1.01, 1.31**	**0.37**	**5.30**
Parity: Multiparous	0.89	0.78, 1.01	0.36	−4.15
Smoking: Yes	**1.25**	**1.08, 1.43**	**0.12**	**2.96**
Diabetes: Yes	1.04	0.83, 1.31	0.04	0.16
Chronic Hypertension: Yes	**1.55**	**1.22, 1.95**	**0.03**	**1.46**
Gestational Hypertension: Yes	**2.89**	**2.54, 3.28**	**0.07**	**11.98**
Eclampsia: Yes	**8.48**	**4.28, 16.77**	**0.001**	**0.67**

*Bolded risk factors are significant at *P* < .05.
